# Reported Safety and Sedation Outcomes of Intranasal Dexmedetomidine as a Sole Sedative for Magnetic Resonance Imaging (MRI) in Children: A Systematic Review and Meta-Analysis

**DOI:** 10.3390/children13060798

**Published:** 2026-06-10

**Authors:** Hossam M. Ajabnoor, Danah Alshihri, Farah Albaqami, Layan Alghamdi, Layla M. Alhazmi, Rana Alghamdi, Alyaa M. Ajabnoor, Rawan O. Almadfaa, Reham M. Baamer

**Affiliations:** 1 Division of Anesthesiology and Intensive Care, Department of Surgery, College of Medicine, University of Jeddah, Jeddah 22425, Saudi Arabia; hajabnoor@uj.edu.sa; 2 Department of Pharmacy Practice, Faculty of Pharmacy, King Abdulaziz University, Jeddah 22254, Saudi Arabia; danahalshihri2@gmail.com (D.A.); farah.m.albaqami@gmail.com (F.A.); layanfahd07@gmail.com (L.A.); layla1422.salman@gmail.com (L.M.A.); rana.ab.alghamdi@gmail.com (R.A.); ralmedfa@kau.edu.sa (R.O.A.); rmbaamer@kau.edu.sa (R.M.B.); 3 Division of Pharmacy & Optometry, School of Health Sciences, Faculty of Biology, Medicine and Health, University of Manchester, Manchester M13 9PL, UK; 4 Division of Pharmacy Practice and Policy, School of Pharmacy, University of Nottingham, Nottingham NG7 2RD, UK

**Keywords:** magnetic resonance imaging, intranasal dexmedetomidine, children, procedural sedation, systematic review

## Abstract

**Highlights:**

**What are the main findings?**

- Across the included observational studies, intranasal dexmedetomidine monotherapy was associated with a high pooled sedation success rate in children undergoing MRI, and most patients did not require rescue sedation.
- Reported hemodynamic adverse events, including bradycardia and hypotension, were uncommon and generally did not require intervention across the included observational studies.

**What are the implications of the main findings?**

- These findings suggest that intranasal dexmedetomidine may be a feasible non-invasive sedation option for children undergoing MRI.
- The substantial heterogeneity across studies highlights the need for prospective studies using standardized methods to better define optimal dosing, patient selection, and outcome reporting.

**Abstract:**

**Background:** Magnetic resonance imaging (MRI) in children may require sedation to minimize motion artifacts and obtain diagnostic-quality images. Intranasal dexmedetomidine (IN DEX) is increasingly used as a non-invasive sedation option; however, evidence regarding its reported effectiveness and safety as a sole agent in routine clinical practice remains limited. This systematic review and meta-analysis aimed to evaluate the reported efficacy and safety of IN DEX monotherapy for pediatric MRI. **Methods:** A systematic search of PubMed, MEDLINE, and Embase via Ovid, Scopus, Web of Science, and the Cochrane Library was conducted for studies published from 17 December 1999 to 10 January 2026. Observational studies involving pediatric patients undergoing MRI with IN DEX as the sole sedative agent were included. Outcomes included sedation success, rescue sedation use, bradycardia, hypotension, sedation onset time, and MRI duration. Random-effects meta-analyses were performed, and methodological quality was assessed using the National Institutes of Health quality assessment tool. **Results:** Twelve observational studies comprising 1828 children were included. The pooled reported sedation success rate was 84% (95% CI: 73–95%), and rescue sedation was required in 19% (95% CI: 8–29%) of cases. The pooled incidences of bradycardia and hypotension were 3% (95% CI: 0–6%) and 1% (95% CI: 0–3%), respectively; no clinically significant events requiring intervention were reported. The pooled mean sedation onset time was 18.4 min, and the pooled mean MRI duration was 38.9 min. Substantial heterogeneity was observed across the efficacy outcomes. **Conclusion:** Intranasal dexmedetomidine appears to be a feasible and well-tolerated option for pediatric MRI sedation. Although pooled observational data suggest high reported sedation success and low adverse-event rates, findings should be interpreted cautiously because of substantial heterogeneity across studies.

## 1. Background

Magnetic resonance imaging (MRI) is widely used in children because it provides high-quality diagnostic images without exposure to ionizing radiation. However, young children may be unable to remain still during the examination because of anxiety, developmental limitations, or lack of cooperation. Patient movement can result in motion artifacts, nondiagnostic images, and the need for repeat imaging [1,2]. Sedation is therefore often required for pediatric MRI; however, it carries risks, including hypoxemia and inadequate or failed sedation [3].

Dexmedetomidine is a highly selective alpha-2 adrenoceptor agonist with sedative, anxiolytic, analgesic, sympatholytic, and opioid-sparing properties [4]. Its sedative profile resembles natural sleep and allows patients to remain arousable and cooperative when stimulated [5]. Compared with sedatives that cause greater respiratory depression, such as opioids and benzodiazepines, dexmedetomidine has relatively limited respiratory effects; however, it may cause bradycardia and hypotension [6,7]. Dexmedetomidine has been used in intensive care and perioperative settings because of its sedative characteristics and potential benefits regarding recovery and emergence agitation [8]. When administered intranasally, dexmedetomidine is rapidly absorbed, with a reported bioavailability of approximately 65% [8]. The intranasal route is particularly attractive in children because it avoids intravenous cannulation for initial administration and may reduce distress and anxiety [9]. Previous studies have reported successful sedation with intranasal dexmedetomidine in children undergoing MRI [10,11].

Previous systematic reviews evaluating intranasal dexmedetomidine (IN DEX) for children undergoing MRI have primarily focused on randomized controlled trials, experimental studies, or single-center studies [12,13,14]. In addition, some reviews evaluated intranasal dexmedetomidine in combination with other sedative agents, such as midazolam or chloral hydrate, rather than examining its use as a sole sedative agent [15]. Consequently, the reported efficacy and safety of IN DEX monotherapy in routine clinical practice remain incompletely characterized. To our knowledge, no previous systematic review and meta-analysis has specifically synthesized observational evidence on IN DEX used as the sole sedative agent for pediatric MRI. Furthermore, safety outcomes, including bradycardia, hypotension, oxygen desaturation, and recovery time, have been reported inconsistently across studies, limiting the comparability of findings. Therefore, this systematic review and meta-analysis aimed to evaluate the reported efficacy and safety of IN DEX as a sole sedative agent for pediatric MRI by synthesizing observational evidence on sedation success and hemodynamic adverse events.

## 2. Methods

This systematic review and meta-analysis was conducted in accordance with the Preferred Reporting Items for Systematic Reviews and Meta-Analyses (PRISMA) 2020 statement. The PRISMA 2020 checklist is provided in Supplementary Table S1 [16]. The review protocol was registered in the International Prospective Register of Systematic Reviews (PROSPERO) under registration number CRD420261278726 [17].

### 2.1. Search Strategy

**Figure 1:**
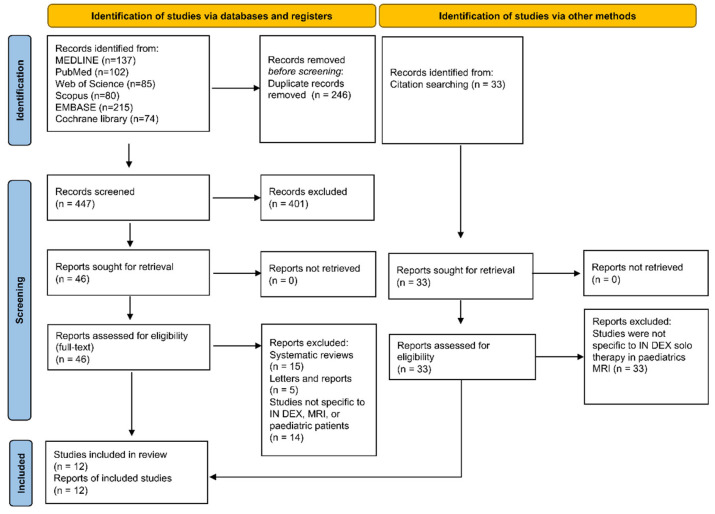
PRISMA 2020 flow diagram summarizing the study-selection process. PRISMA, Preferred Reporting Items for Systematic Reviews and Meta-Analyses.

A comprehensive literature search was conducted in PubMed, MEDLINE, and Embase via Ovid, Scopus, Web of Science, and the Cochrane Library. The search included studies published from 17 December 1999, the date of dexmedetomidine approval by the US Food and Drug Administration, to 10 January 2026. The search strategy combined Medical Subject Headings (MeSH) and free-text terms representing four concepts: (1) dexmedetomidine, (2) intranasal administration, (3) magnetic resonance imaging (MRI), and (4) the pediatric population. These concepts were combined using the Boolean operators “AND” and “OR”; the complete search strategy is provided in Supplementary Table S2. The reference lists of eligible studies and relevant reviews were also manually searched to identify additional eligible studies. No language restrictions were applied, and any non-English studies were translated using both Google Scholar and artificial intelligence-based translation tools. The study-selection process is summarized in the PRISMA 2020 flow diagram shown in Figure 1.

### 2.2. Eligibility Criteria

Studies were eligible for inclusion if they were observational in design, including prospective cohort, retrospective cohort, or cross-sectional studies, and included pediatric patients aged ≤18 years undergoing MRI with IN DEX administered as the sole sedative agent, either in the overall study population or in a separately reported subgroup. Abstracts of observational studies reporting outcomes of interest were also eligible when the full text was unavailable. Studies were excluded if they were case reports, case series, animal studies, randomized controlled trials, or other experimental studies. Observational studies were also excluded if they evaluated interventions intended to prevent adverse effects of IN DEX; used combinations of sedative agents, such as dexmedetomidine with midazolam, ketamine, or chloral hydrate, without a separately reported IN DEX monotherapy group; involved procedures other than MRI; or included surgical procedures or general anesthesia.

### 2.3. Selection of Studies and Data Extraction

Following the database search, all identified records were uploaded to Rayyan systematic review management platform [18], which facilitates systematic-review screening, including blinded assessment and identification of discrepancies between reviewers. Study selection was conducted in two phases. In the first phase, two reviewers independently screened the titles and abstracts of identified records. In the second phase, the same reviewers independently assessed the full texts of potentially eligible studies. Disagreements at either stage were resolved through discussion and consensus, with consultation from a third reviewer when necessary. To ensure objectivity, four reviewers independently extracted data using a prespecified, standardized Microsoft Excel data-extraction form. Extracted data included the study author and year, country, study design, sample size, intervention, comparator, patient age, patient weight, sedation scale, rescue sedation requirement following inadequate IN DEX sedation, type of rescue medication, MRI duration, sedation success rate, adequate sedation, supplemental oxygen requirement, hypotension, and bradycardia, as defined in the individual studies. All extracted data were cross-checked for accuracy and consistency.

### 2.4. Quality Assessment

The methodological quality of the included studies was assessed using the National Heart, Lung, and Blood Institute (NHLBI) Quality Assessment Tool for Observational Cohort and Cross-Sectional Studies, developed by the National Institutes of Health (NIH) [19]. This tool evaluates key aspects of internal validity, including participant selection, exposure and outcome measurement, follow-up, and adjustment for potential confounding. Two reviewers independently assessed each included study and assigned an overall quality rating of good, fair, or poor. Disagreements were resolved through discussion until consensus was reached.

### 2.5. Data Synthesis and Statistical Analysis

The primary outcome was sedation success among pediatric patients who received IN DEX as a sole sedative agent for MRI, as reported as a proportion or percentage in the included studies. Meta-analyses were performed only when at least two studies reported data for a given outcome. The primary quantitative synthesis included all studies reporting sedation success, while secondary analyses evaluated rescue sedation use, bradycardia, and hypotension. When outcome data were missing or unclear, the corresponding authors were contacted for clarification or additional information.

For proportional outcomes, pooled estimates were calculated using a random-effects model with restricted maximum likelihood (REML) estimation to account for between-study heterogeneity. Proportions were transformed using the Freeman–Tukey double-arcsine method to stabilize variance, and pooled estimates were reported with corresponding 95% confidence intervals (CIs). Statistical heterogeneity was assessed using Cochran’s Q test and quantified using the I^2^ statistic, with values of <25%, 25–50%, and >50% considered to indicate low, moderate, and high heterogeneity, respectively. The τ^2^ statistic was also reported as a measure of between-study variance. For continuous outcomes, including sedation onset time and MRI duration, pooled mean estimates were calculated using a random-effects model with REML estimation. Exploratory univariable meta-regression analyses were performed to examine potential sources of heterogeneity in reported sedation success using study-level variables that were consistently available across the included studies. Small-study effects and potential publication bias were explored visually using funnel plots and statistically using Begg’s rank correlation test; these assessments were interpreted cautiously because of the limited number of included studies. All statistical analyses were conducted using Stata version 18.0 (StataCorp LLC, College Station, TX, USA).

## 3. Results

### 3.1. Study Selection

The study-selection process is summarized in the PRISMA 2020 flow diagram shown in Figure 1. A total of 693 records were identified through database searching and manual searching of reference lists. After removal of 246 duplicate records, 447 records remained for title and abstract screening. Of these, 401 records were excluded because they were unrelated to IN DEX or did not involve pediatric MRI sedation. The full texts of 46 reports were assessed for eligibility, of which 34 were excluded because of inappropriate study design, lack of relevant outcome data, or adult-only populations. Ultimately, 12 studies met the eligibility criteria and were included in the qualitative and quantitative syntheses.

### 3.2. Study Characteristics

Twelve observational studies published between 2018 and 2024 were included in this review, comprising 1828 pediatric patients who received IN DEX for MRI sedation (Table 1). Eight studies were described as retrospective or prospective cohort studies, one was cross-sectional in design [20], and the remaining studies used other observational cohort-based designs. Three studies were conducted in Sweden [21,22,23], three in the United Kingdom [11,24,25], two in the United States [20,26], and one each in Singapore [27], Belgium [28], Russia [29], and Italy [30]. Sample sizes ranged from 5 to 1091 participants. The administered IN DEX dose generally ranged from 2 to 4 µg/kg, and some studies permitted an additional dose when initial sedation was inadequate. Most study populations comprised infants and young children, although some studies included children and adolescents up to 18 years of age. Sedation success, image quality, and safety outcomes, including bradycardia, hypotension, and oxygen desaturation, were among the reported endpoints. Brain MRI was the most frequently reported imaging type, while some studies also included spine, extremity, abdominal, pelvic, or internal auditory meatus imaging. Rescue-sedation strategies varied across studies and included additional IN DEX or other sedative agents, such as midazolam, propofol, or chloral hydrate.

### 3.3. The Methodological Quality of the Included Studies

The methodological quality assessment findings are summarized in Table 2. All included studies stated a clear research question and defined their study populations. Eligibility criteria were applied consistently in most studies, although this could not be determined for one study. None of the included studies reported a sample size justification or power calculation, and none reported repeated assessment of exposure. Exposure before outcome assessment and valid exposure and outcome measures were generally documented. Blinding of outcome assessors was either not reported or not performed across the included studies. Adequacy of follow-up was reported in most studies but could not be determined in some. No included study clearly reported adjustment for potential confounding. These methodological limitations should be considered when interpreting the pooled findings.

### 3.4. Results of Meta-Analysis

#### 3.4.1. Sedation Success Rate

**Figure 2:**
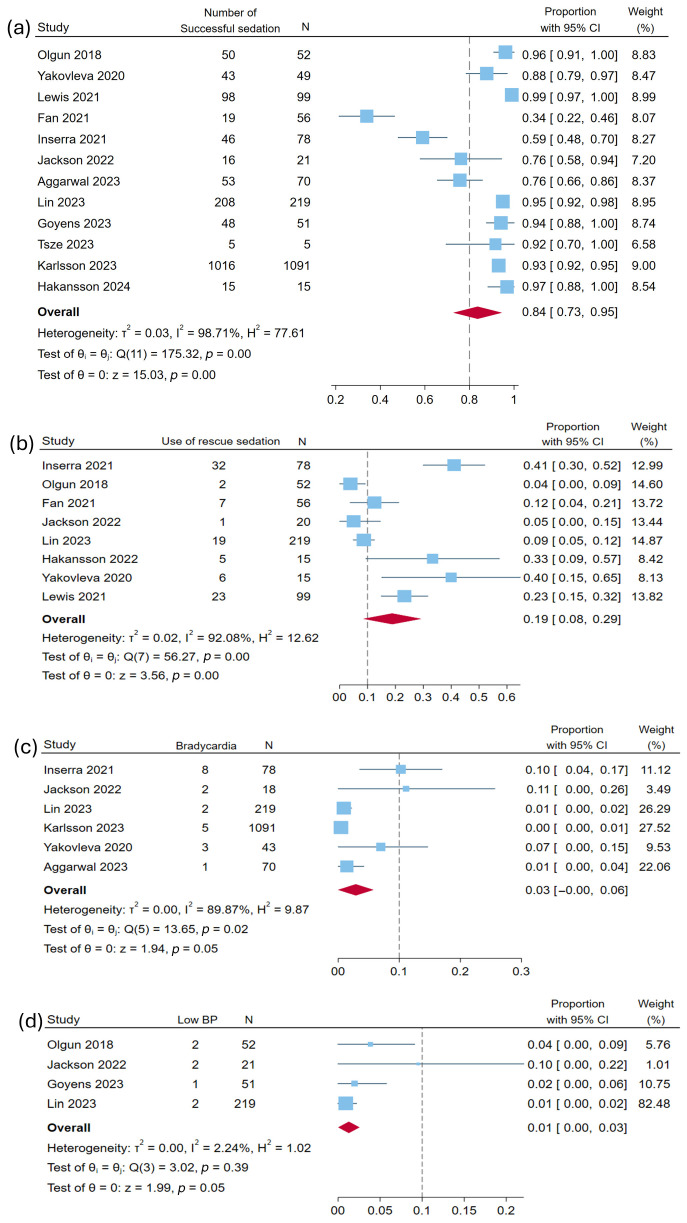
Forest plots of pooled proportional outcomes across the included studies: (**a**) reported sedation success with IN DEX; (**b**) rescue sedation use; (**c**) bradycardia; and (**d**) hypotension. The vertical dashed line is included for visual guidance only and does not indicate a prespecified clinical or statistical cutoff [11,20,21,22,23,24,25,26,27,28,29,30].

All twelve included studies reported the success rate of sedation with IN DEX as a primary outcome, though the definition of “successful sedation” varied across studies (Table 3). Most studies defined success as the completion of MRI without the need for rescue sedation or with diagnostic-quality images. For example, Olgun et al., [26] and Fan et al., [27] considered success the completion of procedures using IN DEX alone, while Inserra (2022) [30], Yakovleva et al., [29] Lin et al., [24] and Karlsson et al., [22] emphasized the acquisition of interpretable or diagnostic-quality images. Across the twelve studies, which included 1,828 children, the pooled success rate of IN DEX sedation was 84% (95% CI: 0.73–0.95) using a random-effects model (Figure 2a). Individual study success rates ranged from 34% for Fan, 2021 [27] to 99% for Lewis, 2021 [21]. Despite the overall high success rate, heterogeneity was substantial (I^2^ = 98.7%, *p* < 0.001), likely reflecting differences in study design, patient age groups, dose regimens, and outcome definitions (Table 1).

#### 3.4.2. Rescue Sedation and Safety Outcomes

Eight studies reported data on rescue sedation or additional sedative dosing following initial IN DEX administration. The pooled proportion of children requiring rescue sedation was 19% (95% CI: 8–29%) using a random-effects model (Figure 2b). Rescue strategies varied across studies and included additional IN DEX doses as well as midazolam, propofol, or chloral hydrate. These interventions were generally administered when initial sedation was inadequate or when patient movement interfered with image acquisition (Table 1). Reported rescue-sedation rates ranged from 4% to 41%, with substantial heterogeneity across studies (I^2^ = 92.08%).

Six studies reported bradycardia outcomes. The pooled reported incidence of bradycardia was 3% (95% CI: 0–6%) (Figure 2c), with individual study incidences ranging from 0% to 11%. Heterogeneity for this outcome was substantial (I^2^ = 89.87%). Four studies reported hypotension outcomes, with a pooled reported incidence of 1% (95% CI: 0–3%) (Figure 2d). No included study reported clinically significant hypotension requiring intervention. Heterogeneity for hypotension was low (I^2^ = 2.24%).

#### 3.4.3. Sedation Onset and MRI Duration

**Figure 3:**
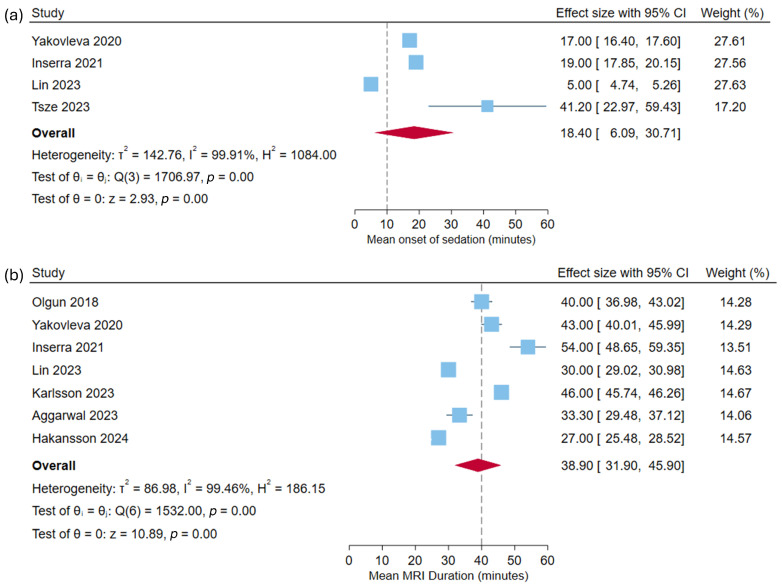
Forest plots of pooled mean outcomes across the included studies: (**a**) sedation onset time following IN DEX administration; and (**b**) MRI duration among children receiving IN DEX sedation. The vertical dashed line is included for visual guidance only and does not indicate a prespecified clinical or statistical cutoff [20,22,23,24,25,26,29,30].

Four studies reported sedation onset time data suitable for quantitative synthesis. The pooled mean sedation onset time following IN DEX administration was 18.4 min (95% CI: 6.09–30.71) using a random-effects model (Figure 3a). Reported mean sedation onset times ranged from 5 min in Lin et al. [24] to 41.2 min in Tsze et al. [20]. Heterogeneity was substantial (I^2^ = 99.91%), indicating marked variability in reported sedation onset times across studies.

Seven studies reported MRI duration data suitable for quantitative synthesis. The pooled mean MRI duration among children receiving IN DEX sedation was 38.9 min (95% CI: 31.9–45.9) using a random-effects model (Figure 3b). Reported mean MRI durations ranged from 27 to 54 min. Heterogeneity was substantial (I^2^ = 99.46%), potentially reflecting differences in MRI protocols, imaging types, patient populations, and sedation pathways across studies.

### 3.5. Meta-Regression and Assessment of Small-Study Effects

Exploratory univariable meta-regression analyses were performed to investigate potential sources of heterogeneity in reported sedation success. The study-level variables examined were MRI type, age category, rescue-sedation protocol, and study sample size, which were selected because they were consistently extractable across the included studies. Given the limited number of included studies and the variability in reporting, these analyses were considered exploratory and were interpreted cautiously. None of the examined variables was significantly associated with the reported sedation success rate (all *p* > 0.05) (Supplementary Table S3). Substantial residual heterogeneity remained in all models (residual I^2^ > 97%), suggesting that the observed between-study variability was not explained by the examined variables and may reflect unmeasured clinical or methodological differences.

**Figure 4:**
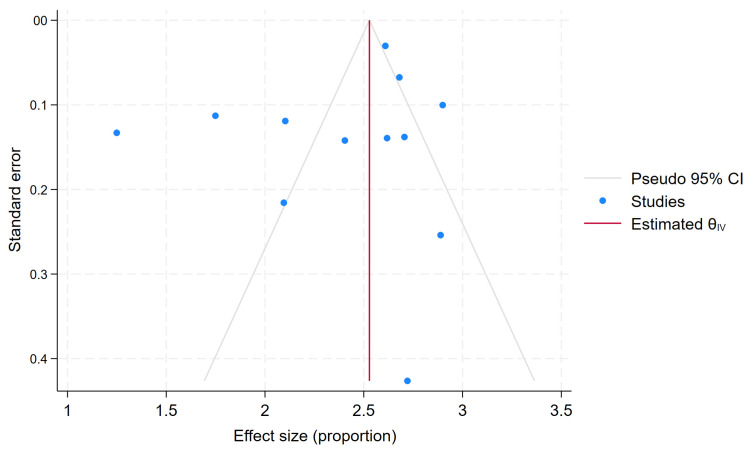
Funnel plot for the primary meta-analysis of reported sedation success with intranasal dexmedetomidine (IN DEX).

Figure 4 presents the funnel plot for the primary meta-analysis of reported sedation success with IN DEX. Visual inspection did not identify marked asymmetry, and Begg’s rank correlation test did not indicate statistically significant funnel plot asymmetry (z = −1.03, *p* = 0.37). However, because only 12 studies were included, the ability of funnel plot inspection and statistical testing to detect small-study effects was limited. Therefore, these findings should not be interpreted as excluding the possibility of publication bias or selective reporting, particularly for adverse events or unsuccessful sedation outcomes.

## 4. Discussion

This systematic review and meta-analysis synthesized observational evidence on IN DEX used as a sole sedative agent for pediatric MRI. Across the included studies, IN DEX was associated with a high pooled reported sedation success rate and low reported incidences of bradycardia and hypotension. However, substantial heterogeneity was observed in sedation success, rescue-sedation use, sedation onset time, and MRI duration. This variability likely reflects differences in patient populations, dosing regimens, MRI protocols, rescue-sedation criteria, and definitions of successful sedation across studies. Most included studies involved infants and young children and were observational in design, with several being retrospective. Therefore, although these findings provide clinically relevant information regarding the use of IN DEX in routine pediatric MRI sedation practice, they should not be interpreted as establishing comparative efficacy or safety relative to other sedation strategies.

Previous systematic reviews and meta-analyses provide important context for interpreting the present findings. A 2022 meta-analysis reported a pooled sedation success rate of 62% (95% CI: 38–82%) for IN DEX monotherapy in children aged 0–8 years undergoing MRI, which was lower than the reported rates for oral chloral hydrate and oral pentobarbital but higher than those for oral or intranasal midazolam and oral triclofos [14]. The same analysis suggested dose-related variation in reported sedation success, with higher doses of IN DEX associated with higher success rates than lower doses [14]. Other comparative reviews have reported that IN DEX may provide greater sedation success than midazolam-based regimens and comparable, although sometimes lower, success than oral chloral hydrate for pediatric MRI sedation [12,14,31]. Regarding safety, previous reviews have generally described IN DEX as having limited respiratory depressant effects, while acknowledging the occurrence of bradycardia and hypotension, which rarely required intervention [12,31]. Some reviews have also suggested that IN DEX monotherapy may be less reliable in older children or during prolonged MRI examinations, whereas its non-invasive administration and generally favorable tolerability remain important potential advantages [12,32].

Evidence from randomized controlled trials (RCTs) and observational studies supports the feasibility of IN DEX monotherapy for pediatric MRI sedation, although reported sedation success rates vary according to study design and clinical context. In a recent three-arm RCT, IN DEX achieved a reported sedation success rate of up to 88% compared with oral triclofos and intranasal midazolam, with no significant adverse events reported [32]. Meta-analyses incorporating RCT data have similarly reported that dexmedetomidine may provide greater sedation success than midazolam and comparable or, in some analyses, greater success than chloral hydrate, with potential advantages in recovery time and adverse-event profiles [14,33,34]. The more standardized dosing protocols and outcome definitions used in RCTs may partly account for their more consistent findings. In contrast, observational studies may better reflect variation in routine clinical practice, including differences in patient characteristics, dosing regimens, MRI protocols, and criteria for rescue sedation. This variability is clinically relevant when considering IN DEX monotherapy for older children or longer MRI examinations, in whom additional sedation may be more frequently required [14,34].

Given these inconsistencies and the lack of MRI-only, monotherapy-focused analyses in the existing literature, our meta-analysis provides a clearer, practice-oriented estimate of the effectiveness and safety of intranasal dexmedetomidine specifically for pediatric MRI—addressing a gap left by prior reviews that combined heterogeneous imaging modalities, mixed drug regimens, and varied study designs. By restricting inclusion to observational studies using intranasal dexmedetomidine as a stand-alone agent for MRI, this review captures real-world sedation performance rather than protocol-driven trial efficacy. The pooled success rate observed across the included studies reflects this pragmatic context, demonstrating that intranasal dexmedetomidine achieves effective sedation for most children, while still revealing a broader range of outcomes than typically reported in RCTs. Likewise, our findings highlight the consistently low incidence of adverse cardiovascular effects, particularly bradycardia and hypotension, across diverse clinical settings, underscoring the favorable safety profile of the drug even when used outside controlled environments. The heterogeneity detected in efficacy outcomes further illustrates the influence of factors such as age, dose variation, and procedural duration, which have been less visible in prior mixed-modality meta-analyses. Exploratory meta-regression analyses using study-level variables such as MRI type, age category, rescue-sedation protocol, and sample size did not significantly explain the observed heterogeneity in our analysis, suggesting that variability was likely multifactorial and influenced by unmeasured clinical and methodological differences across studies. Together, these results provide clinicians with more applicable evidence for decision-making in MRI sedation pathways and help contextualize where intranasal dexmedetomidine performs reliably and where additional adjunctive sedation may be anticipated.

Existing randomized trials and large observational studies consistently report a low incidence of adverse events associated with intranasal dexmedetomidine monotherapy, with transient bradycardia, mild hypotension, and prolonged sedation being the most commonly described effects, typically resolving without intervention [14,28,35,36]. Respiratory safety has been a particular strength of intranasal dexmedetomidine, with large studies reporting very low rates of oxygen desaturation and no life-threatening respiratory complications, supporting its use in noninvasive pediatric procedures [14,28,37]. Cardiovascular effects, including bradycardia and hypotension, have been reported more frequently in certain populations, such as preterm infants, but these events have generally been mild and self-limiting, even in randomized controlled settings [14,37]. Although long-term neurodevelopmental safety data in humans remain limited, no clinical studies to date have demonstrated serious long-term adverse outcomes following intranasal dexmedetomidine exposure [14,32,37]. The findings of this present meta-analysis align closely with this established safety profile. Across the included MRI-focused observational studies, the pooled incidence of bradycardia and hypotension remained low, with events described as clinically mild and not requiring pharmacologic treatment or interruption of imaging. Consistent with prior large studies, respiratory adverse events were uncommon, and no studies reported severe hypoxemia or the need for airway intervention. Notably, the safety outcomes observed in this review reflect real-world clinical practice, encompassing a broad range of patient ages, dosing strategies, and imaging protocols, thereby reinforcing the external validity of earlier trial-based evidence. Taken together, these results confirm that intranasal dexmedetomidine monotherapy for pediatric MRI sedation maintains a favorable cardiovascular and respiratory safety profile comparable to what was reported in controlled trial settings.

From an implementation perspective, the observed variability in sedation success, rescue-sedation use, and reported adverse events highlights the importance of standardized monitoring and reporting frameworks for pediatric MRI sedation. Existing pediatric procedural sedation guidance emphasizes that sedation is a continuum and that monitoring should be sufficient to detect unintended deep sedation, airway compromise, and cardiovascular instability early [35,36,37]. Core monitoring elements generally include continuous clinical observation of airway, breathing, circulation, and level of consciousness, continuous pulse oximetry with heart rate display, and regular documentation of blood pressure and respiratory rate [35,36,38]. Capnography is also increasingly recommended where available, particularly during moderate-to-deep sedation, as an additional safety measure for early detection of hypoventilation [36,37]. In addition to physiologic monitoring, future studies should use standardized definitions of procedural success, such as completion of MRI with diagnostic-quality images without unplanned escalation of sedation, alongside clearly defined rescue criteria and age-appropriate thresholds for bradycardia and low BP. Such standardization would improve comparability across studies and provide clinicians with more reliable evidence for implementing IN DEX sedation pathways in routine pediatric imaging practice.

This meta-analysis has several limitations that should be acknowledged. First, although observational studies provide valuable insight into real-world clinical practice, they inherently limit causal interpretation and are susceptible to selection bias, residual confounding, and inconsistent reporting. In addition, comparative effect estimates for adverse events could not be meaningfully pooled because only a few studies included comparator groups, and the available comparators differed substantially across studies. Therefore, adverse events were synthesized as pooled single-arm incidence proportions, limiting direct comparison between sedation strategies. Second, substantial heterogeneity was observed across most pooled outcomes, likely reflecting differences in patient age distributions, dosing regimens, sedation protocols, MRI types and durations, and definitions of successful sedation. Third, sedation success was not uniformly defined across studies, ranging from completion of MRI without rescue medication to acquisition of diagnostic-quality images, which may have contributed to variability in pooled estimates. Fourth, several outcomes—particularly sedation onset time, MRI duration, and adverse events—were reported inconsistently or only in limited subsets of studies, reducing the precision of pooled analyses. Fifth, some studies had small sample sizes, while a few large cohorts contributed disproportionately to the overall dataset, potentially influencing pooled estimates despite the use of random-effects models. Sixth, the included studies primarily evaluated immediate procedural and short-term recovery outcomes, with no systematic assessment of long-term neurodevelopmental or behavioral effects following intranasal dexmedetomidine exposure. Finally, while no significant publication bias was detected, selective outcome reporting cannot be fully excluded, particularly for infrequent adverse events that may be underreported in retrospective studies. Also, unpublished or ongoing studies not captured in the published observational literature may exist, potentially contributing to publication or reporting bias.

## 5. Conclusions

This systematic review and meta-analysis suggests that intranasal dexmedetomidine, when used as a sole sedative agent, is a feasible and generally well-tolerated option for pediatric MRI sedation. Across the included observational studies, IN DEX was associated with a high pooled reported sedation success rate and low reported incidences of bradycardia and hypotension. However, the substantial heterogeneity across outcomes and the observational nature of the available evidence require cautious interpretation of these findings. Because the included evidence predominantly reflects infants and young children, the applicability of the findings to older children and adolescents remains uncertain. Future prospective studies using standardized dosing regimens, definitions of sedation success, rescue-sedation criteria, and safety-outcome reporting are needed to better define the role of IN DEX monotherapy in pediatric MRI sedation.

## Figures and Tables

**Table 1:** Characteristics of included studies.

Author, Year, Country	Study Design and Study Period	Sample Size	Intervention	Comparator	Patient Age	Inclusion Criteria	Exclusion Criteria	MRI Type	Reported Outcomes	Rescue Sedation/Medication
Olgun 2018 (USA) [26]	Retrospective cohort, from June 2014 to December 2016	52	IN DEX 4 µg/kg	NR	5–8 months	Infants 1–12 months undergoing non-contrast MRI	Other sedatives; assisted ventilation	Brain; spine; extremity	Sedation success; need for rescue medication	Propofol
Yakovleva 2020 (Russia) [29]	Retrospective cohort	49	IN DEX 4 µg/kg	Sevoflurane ± midazolam or propofol	5 months–5 years	Patients undergoing neurologic MRI	NR	Brain	Sedation success; WSS = 2; cardiorespiratory AEs	IN DEX 2 µg/kg (additional dose)
Lewis 2021 (Sweden) [21]	Retrospective cohort	99	IN DEX 4 µg/kg	NR	6 months–6 years	Sedation for Brain MRI	NR	Brain	Feasibility; image quality; motion artifacts; rescue need	IN DEX 2 µg/kg (additional dose)
Fan 2021 (Singapore) [27]	Retrospective cohort, from July 2019 to July 2020	56	IN DEX 2–4 µg/kg	NR	8.5–40 months	1 month–18 years; RSS ≥ 3	Invasive procedures; DEX allergy	MRI	Successful completion of sedation (RSS ≥ 3)	IV propofol
Inserra 2021 (Italy) [30]	Observational (2 prospective cohorts and 1 retrospective cohort), from September 2019 to July 2021	78	IN DEX 3 µg/kg	DEX + midazolam or midazolam alone	Neonates, Median + IQR: 14 days (8–23)	Stable term or neonates with indication for MRI	NR	Brain	Safety (bradycardia, desaturation); efficiency (sedation time, scan duration, success rate)	IN midazolam 0.2 mg/kg
Jackson 2022 (UK) [11]	Observational (1 retrospective cohort and 2 prospective cohorts), from February 2019 to May 2021	20	IN DEX 4 µg/kg	Chloral hydrate or oral midazolam	Median + IQR: 40 months (23–49)	Pediatric patients requiring sedation for MRI	Not fasted; unwell; arrhythmia; hepatic/renal impairment; digoxin	Head; spine; forearm	Sedation success rate for MRI	Chloral hydrate
Aggarwal 2023 (UK) [25]	Prospective cohort, from November 2021 to May 2022	70	IN DEX 0.5–4 µg/kg	midazolam or chloral hydrate	Mean ± SD: 4 years old (0.5–11.9 years)	Patients requiring sedation for neuro-MRI	Prior successful sedation elsewhere	Head; IAM; spine; leg/pelvis	Sedation success; AEs; timing; staff feedback	NR
Lin 2023 (UK) [24]	Retrospective cohort, from March 2016 and March 2022	219	IN DEX 4 µg/kg	IV DEX 3 µg/kg bolus + 2 µg/kg/h infusion	Median + IQR: 35 months (22–57)	All patients sedated with DEX for MRI	Cardiac/resp/neurologic disease; digoxin; HTN; apnea; allergy	Brain; spine	Completion rate; sedation/recovery time; AEs	Midazolam
Goyens 2023 (Belgium) [28]	Retrospective cohort, from November 2014 to May 2021	51	IN DEX 3 µg/kg (if <6 months); 4 µg/kg (if ≥6 months)	NR	4 weeks–18 years	ASA I–II children for procedural sedation	ASA ≥ III; acute illness; sleep apnea; cardiomyopathy	Mixed	Efficacy and safety of IN DEX sedation pathway	NR
Tsze 2023 (USA) [20]	Cross-sectional study, from September 2018 to January 2020	5	IN DEX 1–4 µg/kg	Oral or IN midazolam	2 months–17 years	Children requiring sedation for MRI	DEX allergy; hepatic/renal impairment; digoxin; β-blocker	MRI	Sedation success; onset/recovery time; AEs	Oral or IN midazolam
Karlsson 2023 (Sweden) [22]	Retrospective cohort, from December 2017 to May 2021	1091	IN DEX 4 µg/kg	NR	Median + IQR: 34 months (33–36)	ASA I–II children 0–12 years	GA cases; ASA III–IV	Brain; spine; extremities; abdomen MRI	Hemodynamic and respiratory changes before/after IN DEX	IN DEX 2 µg/kg (additional dose)
Hakansson 2024 (Sweden) [23]	Retrospective cohort, from 2020 to 2021	38	IN DEX 4 µg/kg	NR	6 months–5 years	Pediatrics requiring sedation for brain MRI	ASA > II; major comorbidities	Brain MRI	Image quality and service efficiency	NR

Abbreviations: AEs, adverse events; ASA, American Society of Anesthesiologists; DEX, dexmedetomidine; HTN, hypertension; IAM, internal auditory meatus; IN, intranasal; IQR, interquartile range; MRI, magnetic resonance imaging; NR, not reported; RSS, Ramsay Sedation Scale; SD, standard deviation; WSS, Wong Sedation Scale.

**Table 2:** Methodological quality assessment of the included studies using the NHLBI Quality Assessment Tool for Observational Cohort and Cross-Sectional Studies.

Author, Year	Clear Research Question	Defined Population	Participation ≥ 50%	Uniform Selection	Sample Size Justification	Exposure Before Outcome	Adequate Time Frame	Exposure Levels Examined	Exposure Measures Valid	Repeated Exposure Assessment	Outcome Measures Valid	Blinded Outcome Assessors	Low Loss to Follow-Up	Confounders Controlled
**(1) Olgun, 2018** [26]	**+**	**+**	**+**	**+**	**-**	**+**	**+**	**?**	**+**	**-**	**+**	**-**	**?**	**-**
**(2) Yakovleva, 2020** [29]	**+**	**+**	**+**	**+**	**-**	**+**	**+**	**+**	**+**	**-**	**+**	**?**	**+**	**-**
**(3) Fan, 2021** [27]	**+**	**+**	**+**	**+**	**-**	**+**	**+**	**?**	**+**	**-**	**+**	**-**	**+**	**-**
**(4) Inserra, 2021** [30]	**+**	**+**	**+**	**+**	**-**	**+**	**+**	**-**	**+**	**-**	**+**	**?**	**+**	**-**
**(5) Lewis, 2021** [21]	**+**	**+**	**?**	**+**	**-**	**+**	**?**	**-**	**+**	**-**	**+**	**-**	**+**	**-**
**(6) Jackson, 2022** [11]	**+**	**+**	**+**	**+**	**-**	**+**	**+**	**+**	**+**	**-**	**+**	**-**	**+**	**-**
**(7) Goyens, 2023** [28]	**+**	**+**	**+**	**+**	**-**	**+**	**+**	**+**	**+**	**-**	**+**	**-**	**+**	**-**
**(8) Karlsson, 2023** [22]	**+**	**+**	**?**	**+**	**-**	**+**	**+**	**?**	**+**	**-**	**+**	**?**	**+**	**-**
**(9) Lin, 2023** [24]	**+**	**+**	**+**	**+**	**-**	**+**	**+**	**+**	**+**	**-**	**+**	**-**	**?**	**-**
**(10) Tsze, 2023** [20]	**+**	**+**	**?**	**+**	**-**	**+**	**+**	**+**	**+**	**-**	**+**	**-**	**?**	**-**
**(11) Aggarwal, 2023** [25]	**+**	**+**	**?**	**+**	**-**	**+**	**+**	**?**	**+**	**-**	**+**	**-**	**+**	**-**
**(12) Hakansson, 2024** [23]	**+**	**+**	**?**	**?**	**?**	**+**	**+**	**-**	**+**	**-**	**+**	**-**	**+**	**-**

Symbols: (+), green—criterion met; (***-***), red—criterion not met; (?), orange—cannot determine.

**Table 3:** Definitions of sedation success across included studies.

Study	Definition of Successful Sedation
(1) Olgun, 2018 [26]	Completion of the sedation procedure without the need for a rescue drug other than repeat IN DEX
(2) Yakovleva, 2020 [29]	Completion of MRI with diagnostic image quality and without need for additional sedatives or anesthesia
(3) Fan, 2021 [27]	Completion of procedure or investigation with IN DEX as the only agent
(4) Inserra, 2021 [30]	MRI scan was not interrupted due to motion and/or awaking of the neonate or a further dose of either the same or another sedative agent was required.
(5) Lewis, 2021 [21]	Not explicitly defined
(6) Jackson, 2022 [11]	Scans were considered successful if the images were sufficient for a pediatric radiologist to provide a diagnostic opinion
(7) Goyens, 2023 [28]	Successful if no additional intervention (extra rescue dose of dexmedetomidine, 1 μg/kg based on the Ramsay score, or any additional medicine)
(8) Karlsson, 2023 [22]	Acceptable image quality to answer the diagnostic question
(9) Lin, 2023 [24]	Ability to complete the imaging study and/or the ability to obtain images of diagnostic quality, as determined by the consultant radiologist
(10) Tsze, 2023 [20]	Interpretable results associated with IN DEX
(11) Aggarwal, 2023 [25]	Not explicitly defined
(12) Hakansson, 2024 [23]	Not explicitly defined

## Data Availability

The data analyzed in this systematic review and meta-analysis were derived from the published studies included in the review. The extracted data supporting the findings of this study are available from the corresponding author upon reasonable request.

## Supplementary Materials

The following supporting information can be downloaded at: https://www.mdpi.com/article/10.3390/children13060798/s1, Table S1: PRISMA checklist; Table S2: Detailed search strategy; Table S3: Exploratory univariable meta-regression analyses for sedation success rate.
